# The evolution and therapy aspects in multiple organ dysfunction sepsis

**DOI:** 10.1186/1471-2334-13-S1-P86

**Published:** 2013-12-16

**Authors:** Codrina Bejan, Isabela Ioana Loghin, Florin Roşu, Laura Ghibu, Gheorghe Dorobăț, Carmen Dorobăț

**Affiliations:** 1 “Gr.T. Popa” University of Medicine and Pharmacy, Iaşi, Romania; 2 Clinical Hospital of Infectious Diseases “Sf. Parascheva”, Iaşi, Romania; 3 “N. Oblu” Clinical Hospital of Neurosurgery, Iaşi, Romania

## Background

Severe sepsis was the subject of intense debate over the years, and in the last twenty years there has been a constant effort to identify therapies that reduce mortality. Although the inflammatory response is often adapted to the etiologic agent and to the increase of the mediators in association with organ dysfunction, it is not currently possible to rapidly assess the patients’ ability to develop an appropriate response.

## Methods

We conducted a retrospective study over a period of 7 months, January to July 2013, in which we included 90 patients with primary diagnosis of sepsis, admitted to the Clinical Hospital of Infectious Diseases “Sf. Parascheva”, Iaşi, where we evaluated the following parameters: clinical, etiological, lab results, Carmeli prognostic score, APACHE II score, antibiotherapy. We tried to identify correlations and statistically significant differences. The data were processed using 16.0. SPSS version.

## Results

No statistically significant difference was identified between the ages of patients who had two or three associated organ dysfunction compared to those with uncomplicated sepsis (p=0.292). Etiologic agents could be established in only 16 cases, organic response and systemic affection were varied, and we did not identify correlations between inflammatory syndrome parameters, regardless of the Carmeli or APACHE II prognostic score group, or identification of the etiologic agent. Antibiotherapy in double association (84 cases) has decreased the fever and periods of hospitalization, and we observed no statistically significant difference compared to the cases in which the triple therapy was used.

## Conclusion

More than two thirds of patients were assigned in group 2 and 3 of Carmeli and APACHE II scores (79 and 74 patients). We identified correlations between the high values of glucose and the presence of organ dysfunction in sepsis in studied patients. No statistically significant difference was found regarding the period of decreasing fever, of the hospitalization’s duration in patients with one, two or more organ dysfunctions with or without of identified etiologic agent. No correlations were observed between the development of sepsis severity (patients with APACHE II and III) and the presence of anemia or thrombocytopenia, or between fever syndrome and inflammatory syndrome.

